# A commentary on “Antipsychotic-induced Parkinsonism is associated with working memory deficits in schizophrenia-spectrum disorders”

**DOI:** 10.3389/fnbeh.2015.00131

**Published:** 2015-05-27

**Authors:** Amir Salem, Ahmed A. Moustafa

**Affiliations:** ^1^School of Social Sciences and Psychology, University of Western SydneySydney, NSW, Australia; ^2^Department of Veterans Affairs, Marcs Institute for Brain and Behaviour, University of Western SydneySydney, NSW, Australia

**Keywords:** schizophrenia, parkinsonism, working memory, cognitive flexibility, negative symptoms

In a recent study, Potvin et al. (2014) used a multivariate statistical approach to identify socio-demographic, neurological, drug induced extrapyramidal symptoms (EPS), and psychiatric predictors of cognitive impairments in patients with a DSM-IV diagnosis of schizophrenia (aged 18–60). Specifically, the study focused on antipsychotic-induced EPS, such as Parkinsonism which is characterized by hypokinesia, tremors, muscle rigidity, and/or bradykinesia (Tandon and Jibson, 2002). EPS were assessed with the Extrapyramidal Symptoms Rating Scale (ESRS; Chouinard and Margolese, 2005) and neurocognition was measured using the Cambridge Neuropsychological Test Automated Battery (CANTAB, Eclipse version 2.0; Elliott et al., 1998). The depressive and psychiatric symptoms of schizophrenia were evaluated using the Calgary Depression Scale for Schizophrenia (CDSS; Addington et al., 1992) and the Positive and Negative Syndrome Scale (PANSS; Kay et al., 1987).

Potvin et al. (2014) found an association between antipsychotic-induced Parkinsonism and working memory deficits in schizophrenia. Further, it was found that negative symptoms were associated with increased deficits in cognitive flexibility, working memory, planning, and visual paired association learning in schizophrenic patients (Elliott et al., 1998). Socio-demographic variables were also found to be significantly related to cognitive impairment in schizophrenia patients. These variables included the number of hospitalizations, age, gender, and education level (Potvin et al., 2014). Prior studies support these findings as it is known that lower SES are correlated with lower education levels and riskier health behaviors.

It should be noted, however, that while SES was shown by Shah et al. (2012) to be correlated with cognitive impairments in dementia patients and by Potvin et al. (2014) in schizophrenia patients, education or income variation alone may not be the reason why low SES is correlated with cognitive impairments in clinical patients. It could be the differing life choices, occupations and environments resulting in different levels of propensity across SES groups to experience life stresses and suffer from chronic or acute illnesses (Schurer et al., 2014). However, it cannot be discounted that SES factors could simply be comorbid factors with no causal or contributing links to cognitive impairments in schizophrenia patients. Further research is needed to find more definitive causal links or at least diminish SES factors as confounding variables in understanding drug induced EPS in schizophrenia patients. These results were found using multiple hierarchical linear regression analyses which explored the associations between drug induced EPS and cognition, while taking into consideration the effect of socio-demographic and psychopathology variables.

The Potvin et al. (2014) results are in agreement with previous studies, suggesting a small-to-moderate association between negative symptoms (but not positive symptoms) and cognitive impairments in schizophrenia patient. These cognitive impairments included deficits in problem solving, attention, reasoning, speed of processing, working memory, and verbal/visual memory (Ventura et al., 2009).

Potvin et al. (2014) also posited that the number of hospitalization/duration of illness was found to be associated with cognitive impairments in schizophrenia patients. These results are supported by prior studies (Rajji and Mulsant, 2008; Shah et al., 2012). In the Potvin et al. study, males outperformed females across the three groups: schizophrenia patients (*N* = 218), unaffected first degree subjects (*N* = 438), and healthy non-relatives volunteers (*N* = 123), which in agreement to a cross-sectional study conducted by Torniainen et al. (2011).

Importantly, Potvin et al. (2014) found an association between antipsychotic-induced Parkinsonism and working memory deficits in schizophrenia patients. These are consistent with a study conducted by Jellinger (2010) on Parkinson's disease patients with no psychotic disorders, showing moderate working memory impairments. Further, Potvin et al.'s suggestion of an association between Parkinsonism and working memory impairments is supported by the literature. This is likely related to frontal lobe deficits in first-episode psychosis patients (Cuesta et al., 2014) and supported by imaging studies showing abnormal prefrontal dorsolateral cortex activations in schizophrenia patients while performing memory tasks (Van Snellenberg et al., 2006). Other studies have also reported a relationship between motor symptoms (e.g., akinesia or difficulty moving) and working memory in Parkinson's disease (Moustafa et al., 2014).

Potvin et al. (2014) study was naturalistic, that is, participants were not intentionally recruit based on increased levels of EPS or for specific symptoms (positive or negative). Rather, patients were recruited based on their clinical diagnosis, which makes the association of drug induced Parkinsonism and memory impairment in schizophrenia patients notable considering memory deficits are generally considered as an endophenotype of schizophrenia. While generally speaking schizophrenia endophenotypes can lead to occupational and social impairments, cognitive, specifically working memory, deficits, are one of the leading known causes of impairment in occupational and social functioning in schizophrenia patients (Bowie and Harvey, 2005; Barch et al., 2012).

Regardless of SES, prescribing antipsychotics is a common practice when treating for schizophrenia, even with the well-documented range of side effects, which include but is not limited to weight gain, hypotension, sedation, sexual dysfunctions, emotional instability, and EPS. Further, first-generation antipsychotics (FGAs), such as chlorpromazine and haloperidol, are more likely than second-generation antipsychotics (SGAs), such as Clozapine and Olanzapine, to produce EPS (Leucht et al., 2013). Additionally, a meta-analysis conducted by Leucht et al. (2013) on 15 different antipsychotics (including, Olanzapine, Clozapine, Risperidone, Chlorpromazine, Zotepine, Asenapine, and Haloperidol) had shown that different antipsychotics had varying effect sizes and ranges on patients' experience of EPS. Importantly, some studies found that first-generation (typical) antipsychotics, such as haloperidol, have higher affinity to D2 receptors and more associated with Parkinsonism than second-generation (atypical) antipsychotics, such as clozapine (Abi-Dargham and Laruelle, 2005; Joy et al., 2006; Juckel et al., 2006). It is also important to note that some studies found that some typical antipsychotics may similar effects to atypical antipsychotics on the brain and cognition and that some atypical antipsychotics are difference from each other, suggesting that the differences among these drugs can be related to their pharmokinetics, rather than being typical vs. atypical drug (Kapur et al., 1999).

As such, the results of Potvin et al. (2014) may be affected by lacking some experimental control in relation to the dosage and comparing prescribed antipsychotics. This could be rectified by calculating and substituting a chlorpromazine equivalent for participants in this study to rule out any confounding dosage and drug variations. Further, using unaffected relatives and healthy control group for comparative data of performance could have provided further insight on drug induced Parkinsonism in schizophrenia patients.

## Conflict of interest statement

The authors declare that the research was conducted in the absence of any commercial or financial relationships that could be construed as a potential conflict of interest.
